# Selection and evaluation of reference genes for expression analysis using quantitative real-time PCR in the Asian Ladybird *Harmonia axyridis* (Coleoptera: Coccinellidae)

**DOI:** 10.1371/journal.pone.0192521

**Published:** 2018-06-11

**Authors:** Cheng Qu, Ran Wang, Wunan Che, Xun Zhu, Fengqi Li, Chen Luo

**Affiliations:** 1 Institute of Plant and Environment Protection, Beijing Academy of Agriculture and Forestry Sciences, Beijing, China; 2 Department of Pesticide Sciences, Shenyang Agricultural University, Shenyang, China; 3 State Key Laboratory for Biology of Plant Disease and Insect Pests, Institute of Plant Protection, Chinese Academy of Agricultural Sciences, Beijing, China; CSIRO, AUSTRALIA

## Abstract

*Harmonia axyridis* (Coleoptera: Coccinellidae) is a polyphagous insect that is an important biological agent used to control agricultural and forestry pests. The role of functional genes in *H*. *axyridis* based on quantitative real-time PCR (qRT-PCR) is increasingly well understood to investigate biology, physiology, feeding behavior and the role of important genes in physiological processes. Quantitative real-time PCR (qRT-PCR) is a powerful and reliable technique to quantify gene expression. Using qRT-PCR, expression levels of target genes are determined based on the levels of internal reference genes; therefore, reference genes need to be stably expressed under specific experimental conditions. However, there have been no studies on the stability of reference genes used in *H*. *axyridis*. In this study, we systematically investigated expression profiles of nine candidate reference genes from *H*. *axyridis*, including β-actin (*ACTIN*); elongation factor 1 α (*EF1A*); ribosomal proteins L10, L18, L28, S13, and S15 (*RPL10*, *RPL18*, *RPL28*, *RPS13* and *RPS15*); glyceraldehyde-3-phosphate dehydrogenase (*GAPDH*); and superoxide dismutase (*SOD*). Four analytical methods (geNorm, BestKeeper, NormFinder, and the ΔCt method) were used to evaluate the suitability of these genes as internal reference genes for three biotic factors (developmental stage, tissue, and sex) and two abiotic treatments (temperature and photoperiod). RefFinder, a comprehensive evaluation platform integrating the four analytical methods, was used to rank the overall stability of these reference genes. Among the nine candidate genes, different reference genes were identified as having the most stable expression across biotic and abiotic factors. Genes encoding ribosomal proteins typically had the most stable expression, though *EF1A* was the most stable across developmental stages and photoperiods. To validate the suitability of these reference genes, heat shock protein 90 (*HSP90*) was chosen as a target. Significant up-regulation in *HSP90* expression level in response to both low and high temperature was observed when using the most suitable reference genes but not when using an arbitrarily selected reference gene. The reference genes identified in this study will provide the basis for future functional genomics research in *H*. *axyridis* and will also facilitate the establishment of a standardized qRT-PCR program for other related insects.

## Introduction

*Harmonia axyridis* (Coleoptera: Coccinellidae) is a polyphagous insect that is native to northeast Asia and has been widely introduced as a biological control against pest aphids. Unfortunately, it has unintentionally spread to many other countries [1]. The role of functional genes is increasingly well understood, allowing for them to be used in species like *H*. *axyridis* to investigate biology, physiology, and feeding behavior [2–5], as well as the role of important genes in physiological processes [6]. In addition, the identification of functional genes involved in host-parasite interactions is an important next step in understanding population dynamics [7].

Quantitative real-time PCR (qRT-PCR) is a reliable and reproducible method for gene quantification [8]. Although qRT-PCR has become one of the most-used techniques in molecular biology research, analysis of gene expression is limited by the integrity and quality of RNA samples, reverse transcription, normalization, and PCR efficiency [9, 10]. The typical method for normalizing gene expression data is to simultaneously measure the expression levels of one or more reference genes (also called an endogenous control or housekeeping genes), which are involved in basic and ubiquitous cellular functions and typically exhibit stable and constitutive expression levels across various biotic and abiotic conditions [8]. There are many reference genes that have been widely used for the normalization of qRT-PCR data, such as ribosomal protein, β-actin (*ACTIN*), elongation factor 1 α (*EF1A*), glyceraldehyde-3-phosphate dehydrogenase (*GAPDH*), and superoxide dismutase (*SOD*) [11–15]. Because reference genes are necessary for survival, it is often thought that there is little fluctuation in the transcription of these genes. For example, ribosomal proteins are important components of ribosomes and play important roles in intracellular protein biosynthesis, cell differentiation, and DNA repair, among other functions [16]. EF1A plays an important role in translation by catalyzing the GTP-dependent binding of aminoacyl-tRNA to the acceptor site of the ribosome [17]. SOD, which is a metalloenzyme capable of catalyzing the dismutation of the superoxide anion to elemental molecular oxygen and hydrogen peroxide, is widely distributed in insects [18]. ACTIN is a major component of the cellular skeleton, which maintains structural integrity and gives shape to cells [17]. GAPDH plays a role in energy metabolism [19]. However, many studies have demonstrated that these widely used reference genes are differentially expressed under different experimental conditions [17, 20]. Differentially expressed genes could usually make significantly biological changes across various tissues, developmental stages, sex, and other samples from diverse experimental conditions. Thus, it is important to identify genes with stable expression so that specific changes in gene expression can be evaluated.

Some recent studies have attempted to determine the stability of reference genes in arthropods [15, 21–23]. Yang et al. [24, 25] and Pan et al. [26] identified stable reference genes in three species of ladybeetles: *Coleomegilla maculate*, *Coccinella septempunctata*, and *Hippodamia convergens*. However, there are no available data on the most appropriate genes to use for *H*. *axyridis* target gene normalization under different conditions and at varied developmental stages.

The objectives of this study were to identify appropriate *H*. *axyridis* reference genes for real-time qRT-PCR experiments under various conditions and to provide the basis for future functional genomics research in *H*. *axyridis*. Nine commonly used reference genes, including *ACTIN*, *EF1A*, ribosomal proteins L10, L18, L28, S13, and S15 (*RPL10*, *RPL18*, *RPL28*, *RPS13*, and *RPS15*), *GAPDH*, and *SOD* from *H*. *axyridis* were tested. The effectiveness of these genes for expression normalization was further validated by qRT-PCR analysis of the well-studied target heat shock protein 90 (*HSP90*) gene. Many studies have demonstrated that heat-shock proteins act as molecular chaperones and play an important role in cellular responses to environmental stressors, including sublethal heat and cold shocks, infections, environmental contaminants [6, 27–29]. *H*. *axyridis* is the focus of agriculture and forestry production pest control strategies worldwide. And studying the temperature adaptability of *H*. *axyridis* is very important. Thus, it is of interest to study HSPs like *HSP90* that may confer thermal tolerance. Based on our analysis of *HSP90* and commonly used reference genes, we recommend specific combinations of reference genes to use for qRT-PCR analysis of different biotic and abiotic experimental conditions.

## Materials and methods

### Insects

*Harmonia axyridis* (Coleoptera: Coccinellidae) was purchased from a commercial company in Beijing (Beijing Kuoye Tianyuan Biological Technology Co., Ltd., http://www.kuoye.com/). *H*. *axyridis* larvae and adults were fed with the aphid *Aphis craccivora* Koch (Hemiptera: Aphididae). Ladybeetles were reared in a growth chamber located at Beijing Academy of Agriculture and Forestry Sciences at a temperature of 23±1°C, with a 16L:8D photoperiod and 70% relative humidity.

### Factors

Effects of the following factors on reference gene expression were measured: development stage, tissue, sex, temperature, and photoperiod. The different developmental stages included eggs (30), first-instar larvae (15), second-instar larvae (10), third-instar larvae (2), fourth-instar larvae (2), pupae (1), and sex (one male and one female adult). The head, midgut, and carcass (body with head and viscera removed) were dissected from fourth instar larvae (10 for each replication) under a microscope (Invitrogen, Carlsbad, CA) and stored in TRIzol reagent. To determine the effect of sex on reference gene expression, one adult female and male were collected separately and placed in 1.5 ml centrifuge tubes. To examine the influence of temperature, groups of two third-instar larvae were separately exposed to 5°C, 20°C, and 35°C for 3 h in a constant-temperature incubator (16L:8D photoperiod, 70±10% RH). To test the effect of photoperiod, groups of two third-instar larvae were kept in a constant-temperature incubator (23±1°C, 70±10% RH) with a 16L:8D, 12L:12D, or 8L:16D photoperiod for 2 d. Each experiment was repeated three times. All samples were quickly frozen in liquid nitrogen after collection and stored at -80°C in 1.5 ml centrifuge tubes for subsequent total RNA extraction.

### Selection of gene sequences and primer design

We selected nine housekeeping genes and one target gene from our *H*. *axyridis* transcriptome data: *ACTIN*, *EF1A*, *GAPDH*, *RPL10*, *RPL18*, *RPL28*, *RPS13*, *RPS15*, *SOD*, and *HSP90*. The mfold web server (http://unafold.rna.albany.edu/?q=mfold/DNA-Folding-Form) was used to predict the secondary structure of the DNA template using the following settings: melting temperature, 60°C; DNA sequence, linear; Na+ concentration, 50 mM; and Mg++ concentration, 3 mM. The default settings were used for the remaining parameters [30]. Primers were designed using NCBI Primer-BLAST (https://www.ncbi.nlm.nih.gov/tools/primer-blast/index.cgi?LINK_LOC=BlastHome) with the following settings: GC content between 40–60%; melting temperature of 60°C; and PCR product size between 100–200 base pairs. Excluded regions were defined based on the results of mfold analysis, and the default settings were used for the remaining parameters. PCR primer sequences used for quantification of the ten genes are shown in Table 1.

**Table 1:** Sequences and amplicon characteristics of qRT-PCR primers for nine candidate reference genes and one target gene.

Gene Symbol	Gene Name	Primer sequences (5’-3’)	Length (bp)	Efficiency (%)	R^2^	Linear regression equation
***ACTIN***	B-actin	CTCTTGACCGAAGCCCCATT	114	104.1	0.9996	y = -3.2264x+14.485
		GGAGAGTACGGCTTGGATGG				
***EF1A***	Elongation factor 1 alpha	TCACCGGAACATCTCAAGCC	103	100.6	0.9976	y = -3.3084x+14.218
		GCGTGTTCACGAGTTTGTCC				
***RPL10***	Ribosomal protein L10	AAGGAACCGTAGCCCGAGTA	124	100.4	0.99	y = -3.3084x+14.218
		TTTTGACGACCGGGGAACTT				
***RPL18***	Ribosomal protein L18	AACCCGGTCGTGAAAACCTT	134	97.8	0.9978	y = -3.3765x+14.343
		TGCTTTCACAATTCTGGCACG				
***RPL28***	Ribosomal protein L28	CAGAACCTAGCAACCTCACCA	122	101.7	0.9995	y = -3.2829x+15.442
		AGGTCTCTGACAGACTACGGT				
***RPS13***	Ribosomal protein S13	ACAGACGAAGTGTCCCAACA	135	96.0	0.9988	y = -3.4204x+13.669
		CCTGAGCAACTCCAAGGGAAT				
***GAPDH***	Glyceraldehyde-3-phosphate	GTGAGAGGGATCCCAAAGCC	181	94.7	0.9961	y = -3.455x+17.471
		TCGAGATTGACACCGCAGAC				
***RPS15***	Ribosomal protein S15	CGCCTAGATTCGATGTCCCA	108	100.9	0.9993	y = -3.3002x+14.5
		CCATGATGCCACCGCTAGTA				
***SOD***	Superoxide dismutase	TCAGCTGGAGCACACTTCAA	186	97.3	0.9955	y = -3.3822x+15.218
		ATGTACAACCAGGGTGCGAC				
***HSP90***	Small heat shock protein	TTGGACCCGAAATGCTGACG	145	106.0	0.9988	y = -3.1857x+17.542
		AGGAACAAACAGGAGTGCCC				

### RNA extraction and cDNA synthesis

Total RNA was extracted using the Trizol method. Each sample was homogenized with 1 ml TRIzol reagent (Invitrogen, Carlsbad, CA) following the manufacturer’s protocol. The quality and quantity of RNA were assessed with a Thermo Scientific NanoDrop 2000 UV-Vis spectrophotometer (Thermo Fisher Scientific Inc., Waltham, MA, USA). The quality of the nucleic acid sample was considered good if the OD ratio (A260/A280) was between 1.8 and 2.05 [31]. Complementary DNA (cDNA) was synthesized from 1 μg total RNA using the PrimeScript^™^RT reagent Kit with gDNA Eraser (perfect Real Time) (TAKARA, Japan) according to the manufacturer’s protocol. The cDNA was diluted 10-fold for subsequent qRT-PCR studies.

### qRT-PCR

Real-time qPCR was carried out in 20 μl reactions containing 2.0 μl cDNA, 10 μl SYBR Premix Ex TaqTM II (Takara, Japan), 1 μl forward primer (10 μM), 1 μl reverse primer (10 μM), 0.4 μl Rox Reference Dye II (Takara, Japan) and 5.6 μl nuclease free water using an ABI PRISM 7500 Real-time PCR System (Applied Biosystems, USA). The amplification conditions for qRT-PCR were as follows: 95°C for 30 s, followed by 40 cycles of 95°C for 5 s, and 60°C for 34 s. After all reactions, melting curve analysis (from 60 to 95°C) was done to ensure consistency and specificity of the amplified product. A 10-fold dilution series of cDNA from the whole body of adults was used for a standard curve. The corresponding qRT-PCR efficiencies (E) were calculated using the equation: E = (10^[−1/slope]^ − 1) × 100 [32].

### Constancy analysis of candidate reference genes

The constancy of candidate genes was evaluated using the ΔCT method [33], GeNorm [8], NormFinder [34], and BestKeeper [35]. We also used the online software RefFinder (http://fulxie.0fees.us/?type=reference&ckattempt=1&i=1) to further evaluate the suitability of reference genes by analyzing the results of the four algorithms.

Both geNorm and Normfinder require the conversion of raw cycle threshold (Ct) values into relative quantities. Initially, geNorm calculates a gene expression stability value (M) and then compares the pairwise variation (V) with other genes. Using microarray data as a training set for the algorithm, the pairwise variation between two sequential normalization factors, Vn/Vn+1, is calculated. The optimal number of reference genes required for accurate normalization is determined based on a cutoff Vn/Vn+1 value of 0.15. NormFinder calculates gene expression stability for all samples in any number of groups based on intra- and inter-group variation and combines these values to provide a gene rank order based on the variation in gene expression. BestKeeper uses raw data (Ct values) and PCR efficiency (E) to compute best-suited standards and combines them into an index. The comparative ΔCT method compares the relative expression of pairs of genes within each sample to select the optimal reference gene. Finally, RefFinder evaluates and screens reference genes by integrating the results of the above four major software programs.

### Evaluation of target gene expression

The target gene *H*. *axyridis HSP90* was used to evaluate the performance of nine candidate reference genes. We estimated up- or down-regulation of the *HSP90* gene in *H*. *axyridis* across different temperatures. Relative expression of *HSP90* was calculated using the formula (2^−ΔΔ*C*T^) [36].

## Results

### Evaluation of primer specificity and amplification efficiency

The primer specificity of nine candidate reference genes and one target gene (*HSP90*) was evaluated by PCR. Visualization of PCR products by 2% agarose gel electrophoresis revealed a single amplicon of expected size for each primer pair (S1 Fig). Furthermore, gene-specific amplification was confirmed by a single peak in real-time melting curve analysis (S2 Fig). A 5-point standard with known RNA standard concentrations was used to estimate the amplification efficiencies, which ranged from 94.7% to 106%. The coefficients of all 10 genes based on the linear regression were >0.990 (Table 1).

### Expression profiling of reference genes

**Fig 1:**
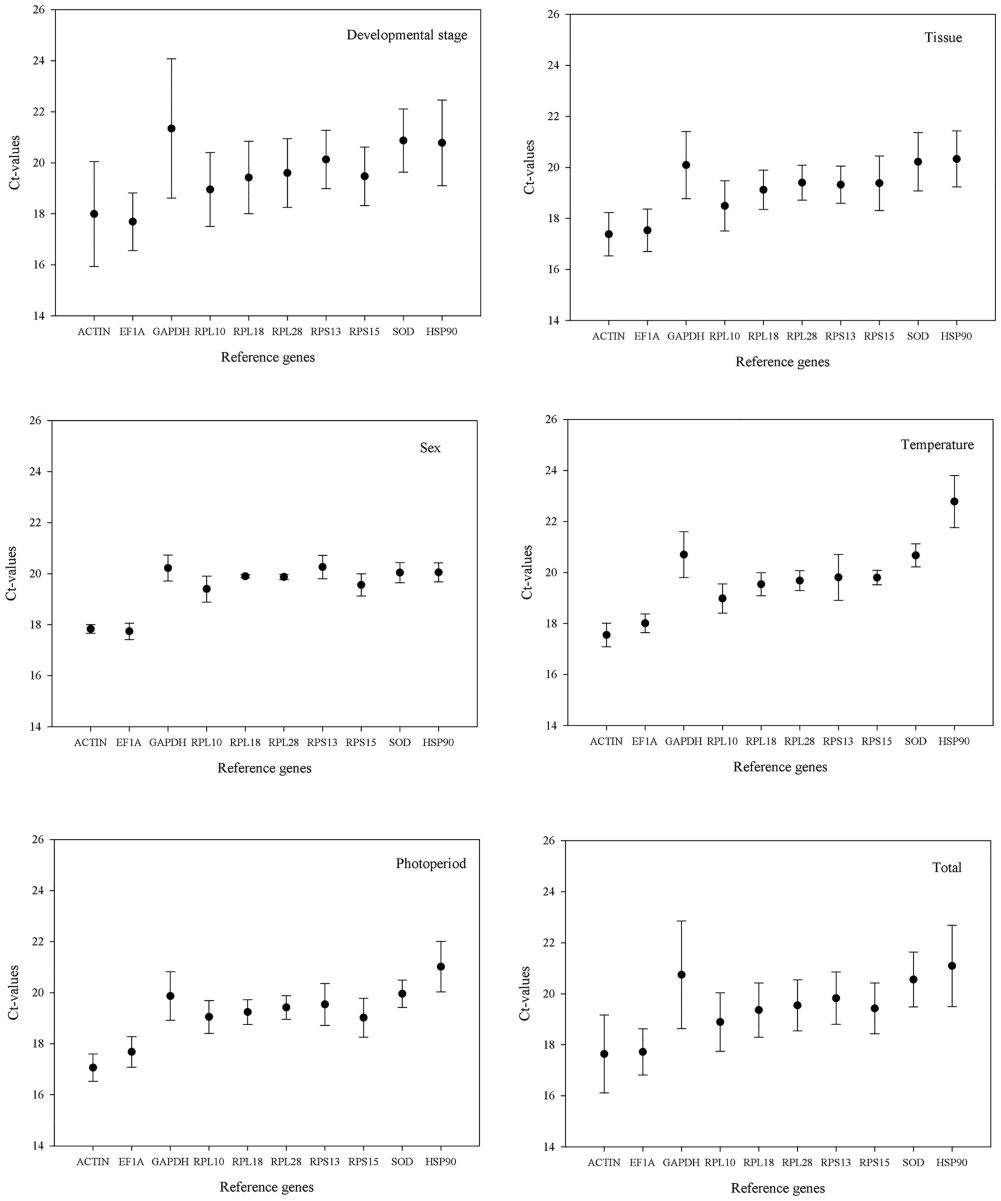
Expression profiles of the nine housekeeping genes and the target gene *HSP90* in *H*. *axyridis* under different factors. Expression level is indicated by cycle threshold (Ct) value. Samples included different development stages, sexes, tissues, photoperiods, and temperatures. Values are means±SD.

Mean Ct values of ten candidate genes of developmental stage, tissue, sex, temperature, photoperiod and total ranged from 17.692 (*EF1A*) to 21.355 (*GAPDH*); 17.384 (*ACTIN*) to 20.325 (*HSP90*); 17.744 (*EF1A*) to 20.258 (*RPS13*); 17.554 (*ACTIN*) to 22.783 (*HSP90*); 17.056 (*ACTIN*) to 21.017 (*HSP90*); 17.640 (*ACTIN*) to 21.094 (*HSP90*). The Ct values of all the tested samples were between 17.056 to 22.783. Among the candidate genes, *ACTIN* and *EF1A* had the highest accumulation of the transcript among different conditions, while *GAPDH* and *HSP90* had the lowest accumulation of transcript levels, and the remaining genes had intermediate transcript expression levels (Fig 1).

### Stability of reference genes across biotic conditions

#### Developmental stage

The most stable genes based on developmental stage were *RPL18*, *RPL28*, and *RPL10* according to *geNorm*. *EF1A*, *RPS13*, and *RPS15* were the most stable according to *Normfinder*, *BestKeeper*, and the *△Ct* method (Table 2).

**Table 2:** Stability of reference gene expression across biotic conditions.

Biotic conditions	Reference genes	*geNorm*		*Normfinder*		*BestKeeper*		*△Ct*	
		Stability	Rank	Stability	Rank	Stability	Rank	Stability	Rank
**Developmental Stage**	*ACTIN*	1.309	7	2.340	8	1.339	7	2.577	8
	*EF1A*	0.710	5	0.099	1	0.910	1	1.209	1
	*GAPDH*	1.619	8	2.506	9	1.981	8	2.705	9
	*RPL10*	0.503	2	0.806	6	1.160	6	1.333	6
	*RPL18*	0.390	1	0.791	5	1.160	6	1.287	4
	*RPL28*	0.390	1	0.784	4	1.083	5	1.291	5
	*RPS13*	0.681	4	0.571	2	0.953	2	1.268	2
	*RPS15*	0.634	3	0.622	3	0.999	3	1.275	3
	*SOD*	0.918	6	1.010	7	1.020	4	1.632	7
**Tissue**	*ACTIN*	0.624	6	0.587	7	0.745	6	0.858	7
	*EF1A*	0.513	3	0.499	4	0.666	4	0.778	5
	*GAPDH*	0.851	8	1.223	9	1.109	9	1.325	9
	*RPL10*	0.533	4	0.507	5	0.730	5	0.768	4
	*RPL18*	0.343	1	0.415	3	0.558	1	0.713	3
	*RPL28*	0.471	2	0.316	1	0.582	2	0.711	2
	*RPS13*	0.343	1	0.404	2	0.620	3	0.704	1
	*RPS15*	0.557	5	0.547	6	0.808	7	0.798	6
	*SOD*	0.715	7	0.790	8	0.848	8	1.001	8
**Sex**	*ACTIN*	0.159	2	0.221	3	0.127	3	0.399	3
	*EF1A*	0.251	3	0.250	4	0.246	4	0.427	4
	*GAPDH*	0.429	7	0.484	8	0.455	8	0.568	7
	*RPL10*	0.395	6	0.428	7	0.493	9	0.532	6
	*RPL18*	0.103	1	0.117	1	0.043	1	0.357	1
	*RPL28*	0.103	1	0.181	2	0.071	2	0.377	2
	*RPS13*	0.467	8	0.526	9	0.420	7	0.600	8
	*RPS15*	0.320	4	0.334	6	0.402	6	0.471	5
	*SOD*	0.362	5	0.331	5	0.279	5	0.471	5

**Fig 2:**
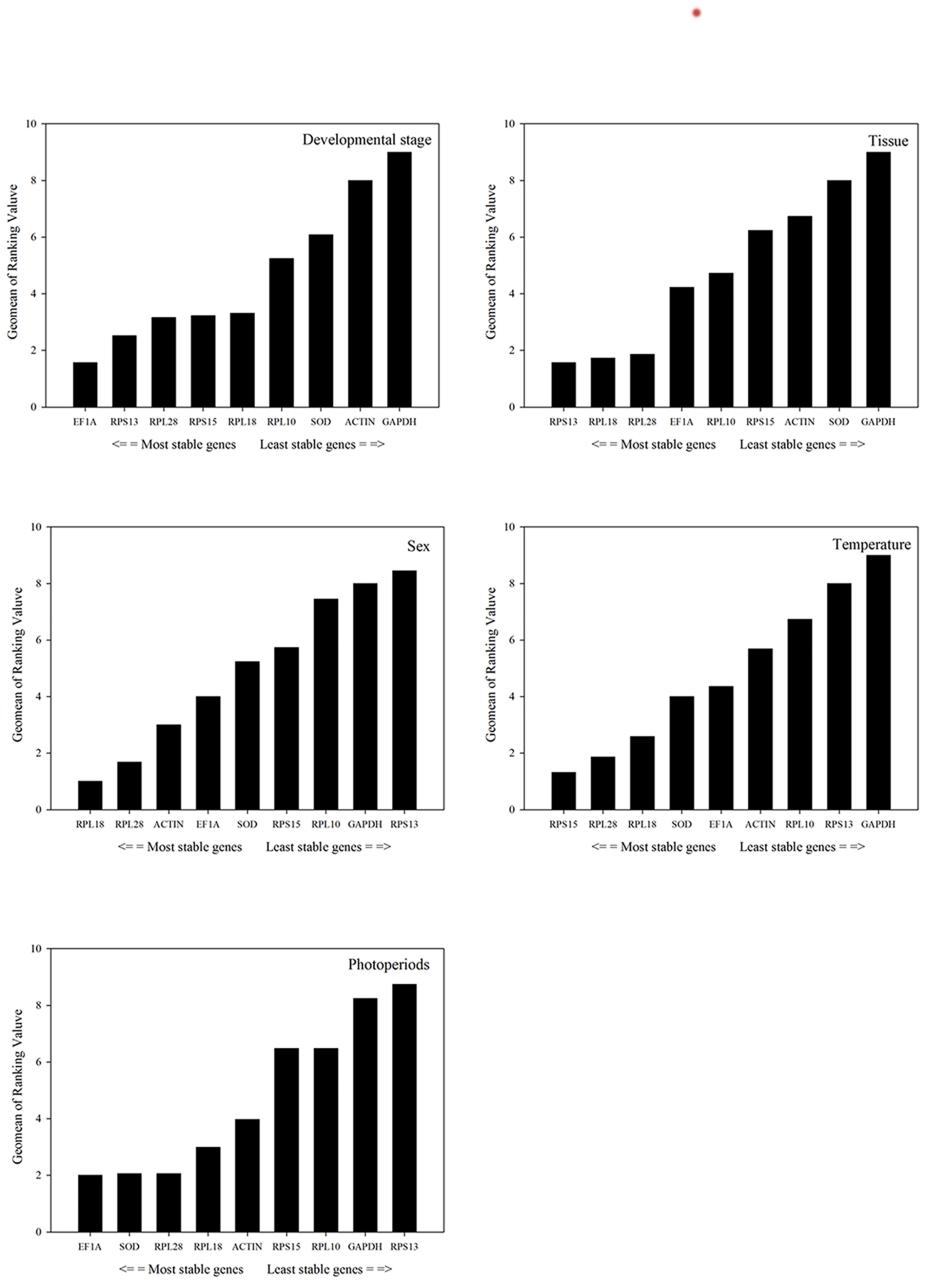
The stability of the expression levels of nine housekeeping genes across different treatments based on *RefFinder* stability value. A lower *Geomean* value indicates more stable expression.

**Fig 3:**
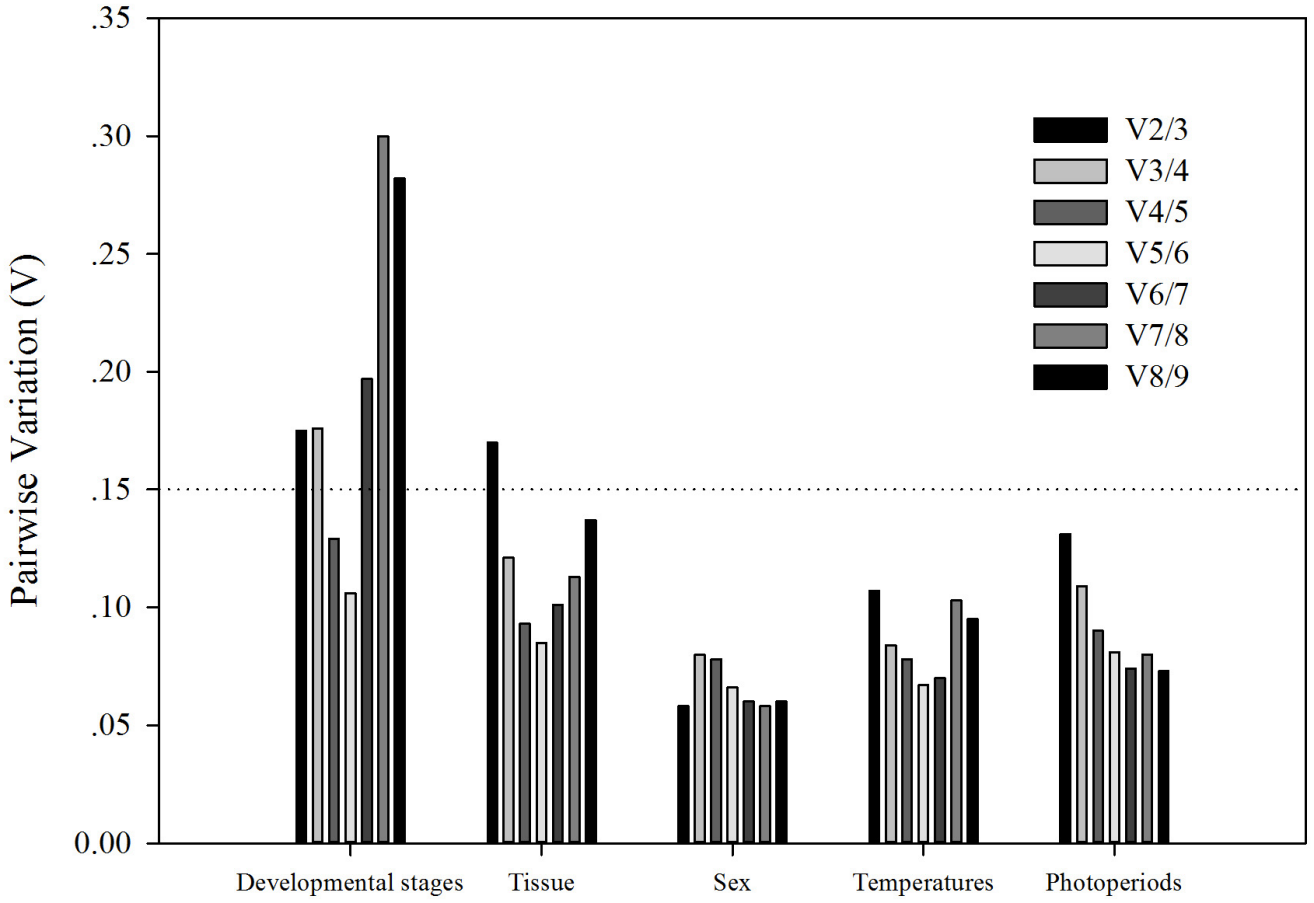
Pairwise variation (Vn/Vn+1) analysis of the candidate reference genes. The pairwise variation was analyzed by GeNorm software. A value <0.15 indicates that normalization would not be dramatically changed by the inclusion of an additional reference gene.

Based on *RefFinder*, the ranking of reference genes from the most to the least stable was: *EF1A*, *RPS13*, *RPL28*, *RPS15*, *RPL18*, *RPL10*, *SOD*, *ACTIN*, and *GAPDH* (Fig 2). From *GeNorm* analysis, the pair-wise values of V2/3 and V3/4 were both above the cut-off value of 0.15 while the pair-wise value of V4/5 was <0.15 (Fig 3). A value <0.15 indicates that supplemental reference genes are unnecessary. Based on *RefFinder* and convenient operation, *EF1A*, *RPS13*, and *RPL28* were determined to be the best reference genes across different developmental stages (Table 3).

**Table 3:** Stability of reference gene expression across abiotic conditions.

Abiotic conditions	Reference genes	*geNorm*		*Normfinder*		*BestKeeper*		*△Ct*	
		Stability	Rank	Stability	Rank	Stability	Rank	Stability	Rank
**Temperature**	*ACTIN*	0.392	4	0.416	6	0.418	7	0.605	5
	*EF1A*	0.42	5	0.415	5	0.265	2	0.616	6
	*GAPDH*	0.646	8	0.840	9	0.737	9	0.934	9
	*RPL10*	0.460	6	0.439	7	0.412	6	0.638	7
	*RPL18*	0.312	1	0.278	3	0.407	5	0.533	3
	*RPL28*	0.312	1	0.247	2	0.318	3	0.519	2
	*RPS13*	0.563	7	0.799	8	0.606	8	0.903	8
	*RPS15*	0.340	2	0.177	1	0.180	1	0.506	1
	*SOD*	0.361	3	0.302	4	0.362	4	0.557	4
**Photoperiod**	*ACTIN*	0.495	4	0.415	5	0.422	2	0.609	5
	*EF1A*	0.472	3	0.258	1	0.429	3	0.535	1
	*GAPDH*	0.595	7	0.629	8	0.725	8	0.755	8
	*RPL10*	0.523	5	0.533	7	0.503	5	0.679	7
	*RPL18*	0.426	1	0.361	4	0.450	4	0.580	4
	*RPL28*	0.426	1	0.354	3	0.429	3	0.572	2
	*RPS13*	0.631	8	0.636	9	0.627	7	0.759	9
	*RPS15*	0.549	6	0.420	6	0.577	6	0.619	6
	*SOD*	0.439	2	0347	2	0.404	1	0.574	3

#### Tissue

Across tissue samples, *RPL18*, *RPS13*, and *RPL28* were the most stable reference genes based on *geNorm*, *Normfinder*, *BestKeeper*, and the *△Ct* method (Table 2), and the ranking of reference gene stability across different tissues based on *RefFinder* was: *RPS13*, *RPL18*, *RPL28*, *EF1A*, *RPL10*, *RPS15*, *ACTIN*, *SOD*, and *GAPDH* (Fig 2). From *geNorm* analysis, the pair-wise value of V3/4 was <0.15 (Fig 3). Thus, *RPS13*, *RPL18*, and *RPL28* were considered the most suitable reference genes for comparisons across different tissues (Table 4).

**Table 4:** Recommended *H*. *axyridis* reference genes for various experimental conditions.

Experimental Conditions	Reference Genes		
**Developmental stage**	*EF1A*	*RPS13*	*RPL28*
**Tissue**	*RPS13*	*RPL18*	*RPL28*
**Sex**	*RPL18*	*RPL28*	
**Photoperiod**	*EF1A*	*SOD*	
**Temperature**	*RPS15*	*RPL28*	

#### Sex

According to *geNorm*, *RPL18*, *RPL28*, and *EF1A* were the most stable reference genes based on sex. *Normfinder*, *BestKeeper* and the *△Ct* method all identified *RPL18*, *RPL28*, and *ACTIN* as the most stable genes across sexes (Table 2).

According to *RefFinder*, the ranking of reference gene stability based on sex was: *RPL18*, *RPL28*, *ACTIN*, *EF1A*, *SOD*, *RPS15*, *RPL10*, *GAPDH*, and *RPS13* (Fig 2). The pair-wise value of V2/3 was <0.15 based on *geNorm* data (Fig 3). Thus, *RPL18* and *RPL28* were considered the most stable reference genes across sexes (Table 4)

### Stability of reference genes under abiotic conditions

#### Temperature

Based on *geNorm*, *Normfinder*, and the *△Ct* method, *RPL18*, *RPL28*, and *RPS15* were the most stable reference genes across temperature treatments. According to *BestKeeper*, *RPS15*, *EF1A*, and *RPL28* were the most stable (Table 3).

According to *RefFinder*, the ranking of reference genes from the most to least stable was: *RPS15*, *RPL28*, *RPL18*, *SOD*, *EF1A*, *ACTIN*, *RPL10*, *RPS13*, and *GAPDH* (Fig 2). The value of V2/3 from *geNorm* analysis was <0.15 (Fig 3). Therefore, *RPS15* and *RPL28* were considered the most stable reference genes across temperature treatments (Table 4).

#### Photoperiod

*EF1A*, *SOD*, and *RPL28* were the most stable reference genes across photoperiod treatments according to *Normfinder* and the *△Ct* method; *RPL18*, *RPL28*, and *SOD* were the most stable according to *geNorm*; and *SOD*, *ACTIN*, *EF1A*, and *RPL28* were the most stable according to *BestKeeper* (Table 3).

According to *RefFinder*, the ranking of reference genes across photoperiod treatments from the most to least stable was: *EF1A*, *SOD*, *RPL28*, *RPL18*, *ACTIN*, *RPS15*, *RPL10*, *GAPDH*, and *RPS13* (Fig 2). The value of V2/3 from *geNorm* analysis was <0.15 (Fig 3). Therefore, *EF1A* and *SOD* were considered the most stable reference genes across photoperiod treatments (Table 4)

### Target gene expression

**Fig 4:**
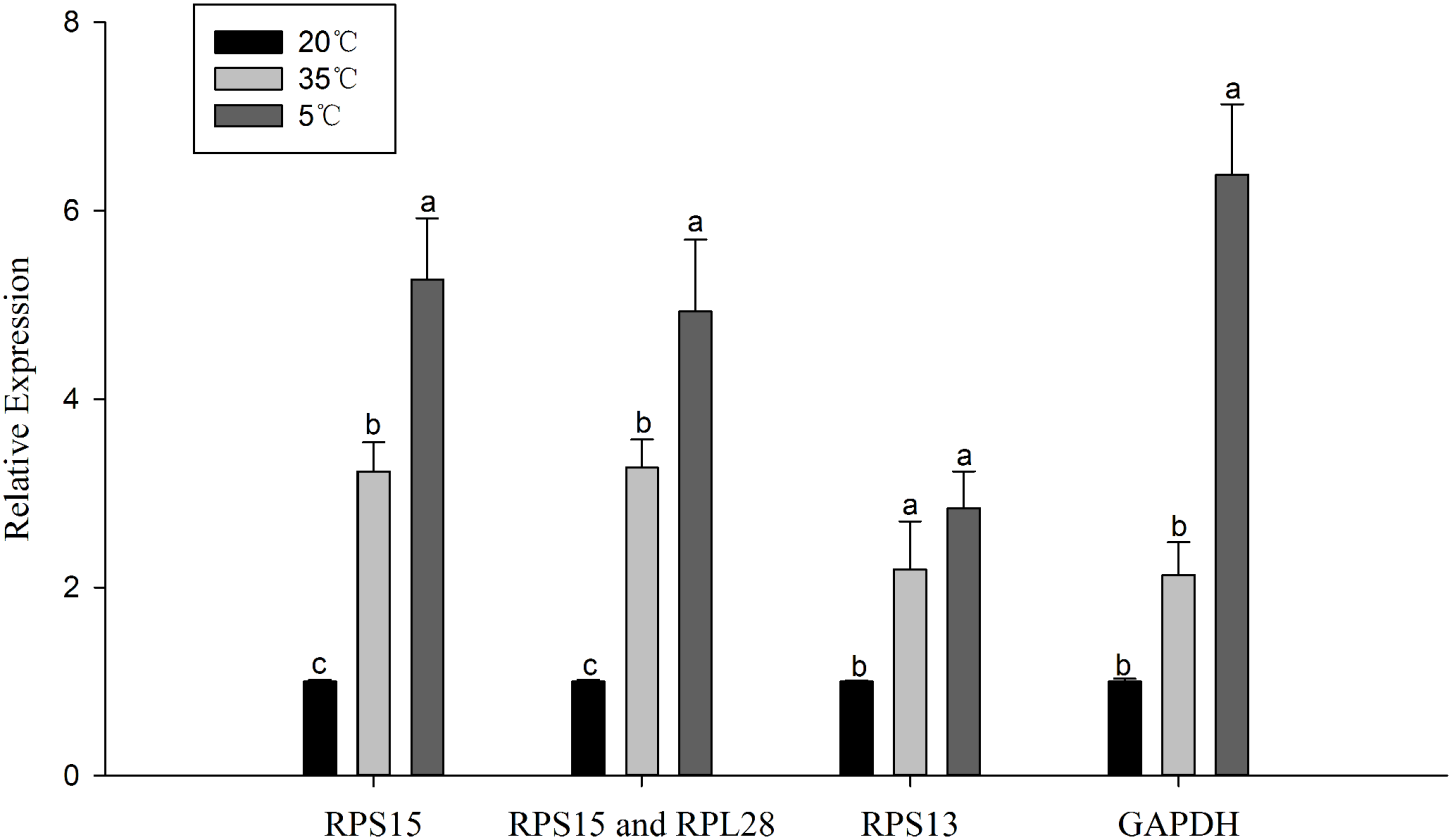
The use of different reference genes to normalize the expression level of the target gene *HSP90* under three temperature treatments was investigated. The expression level was normalized by different candidate reference genes: *RPS15*; *RPS15* and *RPL28*; *RPS13*; and *GAPDH*. The reference genes were selected based on expression stability across three temperature treatments. Data represent the means±SEM of three biological replicates. The comparisons were analyzed using one-way ANOVA, and different letters (a,b) denote significant differences between normalization strategies determined by Tukey test (*P* < 0.05).

To test the effect of reference genes on the calculation of target gene expression, the relative expression level of the target gene *HSP90* under different temperature treatments (5, 20, 35°C) was normalized to the most and least stable reference genes. The relative expression level of *HSP90* significantly differed between temperature treatments. When the most stable reference gene, *RPS15*, was used, *HSP90* was significantly up-regulated under low temperature (5°C) and high temperature (35°C), and the relative expression at 5°C was significantly higher than at 35°C (Fig 4). Similar results were obtained using *RPS13* and *RPL28*. However, the expression level of *HSP90* was not significantly different between the 5°C and 35°C samples when using an unstable reference gene (such as *RPS13*). Similarly, the expression of *HSP90* was not significantly different between 35°C and 20°C samples when the least stable reference gene, *GADPH*, was used (Fig 4).

## Discussion

In the current study, the stabilities of nine reference genes were evaluated across different biotic and abiotic conditions. We found that the best reference genes varied among conditions. The ribosomal proteins typically exhibited the highest stability across different experimental conditions. Across tissues, temperatures, and sexes, *RPS13*, *RPS15*, and *RPL18*, respectively, were the most stable reference genes.

Consistent with our findings, ribosomal proteins have been previously reported to have the highest expression stability in several insects. For instance, in *Bradysia odoriphaga*, expression stability of *RPS15* across temperature treatments was highest [31], and in *Lucilia sericata*, *RPS3* and *RPLP0* showed high stability across specific larval tissues [37]. in *Tetranychus cinnabarinus*, the highest expression stability across tissues was observed for *RPS18* [38]. But expression stability was highest for *RPS11* in *Nilaparvata lugens* [39], and for *RPS13* in *Sesamia inferens* [40]. Our results and the results of previous studies indicate that in general ribosomal proteins are good reference genes for gene expression studies. However, an exception in our study was *RPS13*, which showed the least stable expression across sex and photoperiod treatment. *RPS13* was also found to be the least stable reference gene across photoperiod treatments in *B*. *odoriphaga* [31].

In our study, among the nine reference genes, the expression level of *EF1A* was the most stable across developmental stages and photoperiod treatments. This result is consistent with previous studies concerning developmental stage in *Frankliniella occidentalis* [41] and developmental stage and photoperiod treatment in *Danaus plexippus* (L.) [42].

In our study, *SOD* ranked among the top three reference genes for photoperiod treatments (Table 4). In *Spodoptera exigua*, the expression stability of *SOD* was high across developmental stages and between sexest but was low across certain tissues [15].

*GAPDH* and *ACTIN* have been commonly used as internal controls in many gene expression studies [11, 43, 44]. In our study, however, *ACTIN* ranked low for most factors except for sex, where it was the third most stable gene. Shi et al. (2016) [31] also found that *ACTIN* was a good reference gene in *B*. *odoriphaga* for diet treatments. In *Helicoverpa armigera*, however, *ACTIN* was the least stable reference gene across temperature and photoperiod treatments [45], and in *Tribolium castaneum* exposed to *Beauveria bassiana*, *ACTIN* was not stably expressed [46]. Based on our results, *GAPDH* is also not an ideal reference gene in *H*. *axyridis*. Several studies in other insects have also demonstrated that the expression stability of *GAPDH* is low in some circumstances, such as across developmental stages in *Tetranychus cinnabarinus* [19]; between the labial gland and fat body in *Bombus terrestris* and *Bombus lucorum* [47]; and across different body parts in *Sogatella furcifera* [48]. Taken together, these results suggest that the stability of reference genes may be dependent on the insect species and other characteristics such as instar stage or tissue type.

The ranking of reference genes is not only affected by different experimental conditions or factors, but also by the tools used for ranking. In this study, for example, the most stable reference genes under different temperature treatments were *RPS15*, *RPL28*, and *RPL18* using NormFinder and the △Ct method. BestKeeper also ranked *RPS15* and *RPS28* as the most stable, but *EF1A* was ranked the second most stable. The differences in ranking may result from differences in statistical algorithms. NormFinder and the △Ct method mainly analyze the pairwise variation between reference genes, and then confirm the stability of one gene in each pair. BestKeeper individually analyzes the stability among reference genes [33, 49]. RefFinder, a comprehensive evaluation platform that integrates the four algorithms we tested, was used to estimate the stability rankings of the nine reference genes. We also used GeNorm, which calculates the pairwise variation (Vn/Vn + 1) between the continuous standardization factors or NF (NFn and NFn + 1), to determine the optimal number of reference genes [8]. Based on GeNorm analysis, two reference genes were found to be sufficient for normalizing target gene expression values for sex, photoperiod, and temperature, but three reference genes were needed to normalize across developmental stages and different tissues [Fig 3]. These results suggest that it is necessary to use different combinations of reference genes to study changes in gene expression in *H*. *axyridis* in response to different factors.

In recent years, several reference genes have been used as internal controls for studying gene expression in *H*. *axyridis* under diverse experimental conditions. In this study, we found that nine candidate reference genes have different strengths and weaknesses in various conditions. For example, we recommend *RPS15* and *RPL28* to study gene expression in response to temperature in *H*. *axyridis* to temperature. Previously, Wang et al. [50] selected *RP49* as a reference gene to study the expression of six small heat shock proteins mediating cold-hardiness in *H*. *axyridis*. Moreover, *RP49* was also used as internal control to study the expression of genes in the *H*. *axyridis* trehalase and glycogen metabolic pathways [51, 52]. Vilcinskas et al. [53] also used *RPS3*, another member of ribosomal protein family, as a reference gene in a study of genes encoding antimicrobial peptides and proteins, while study of immunity-related genes was exclusive of our setting conditions. In addition, Tang et al. [54] selected another 18s RNA to verify the expression of cold-resistance response genes, such as E3 ubiquitin-protein ligase, transketolase, trehalase, serine/arginine repetitive matrix protein 2, glycerol kinase and sugar transporter SWEET1-like. One of the most frequently used reference genes, *ACTIN*, was used as the internal control to study development-related genes in the ovaries of adult *H*. *axyridis* [55], and our study showed that this gene may not be as good as other reference genes for some factors such as developmental stage, tissue, temperature, and photoperiods.

Taken together our results indicate that commonly used reference genes are often not well-suited for normalization in all qRT-PCR experiments, and the simultaneous measurement of a panel of candidate reference genes is critical for the accuracy of qRT-PCR quantification. The results of our study represent an important step for establishing a standardized gene analysis framework for *H*. *axyridis*.

## Supporting information

S1 Fig The agrose gel electrophoresis of the nine reference genes. (TIF)

S2 Fig Melting curves of the nine candidate reference genes. (TIF)
