# Supplementary figures and images for: Selection and evaluation of reference genes for expression analysis using quantitative real-time PCR in the Asian Ladybird Harmonia axyridis (Coleoptera: Coccinellidae)
Source: PLoS One. 2018 Jun 11;13(6):e0192521. doi: 10.1371/journal.pone.0192521 (PMC5995347; doi:10.1371/journal.pone.0192521)

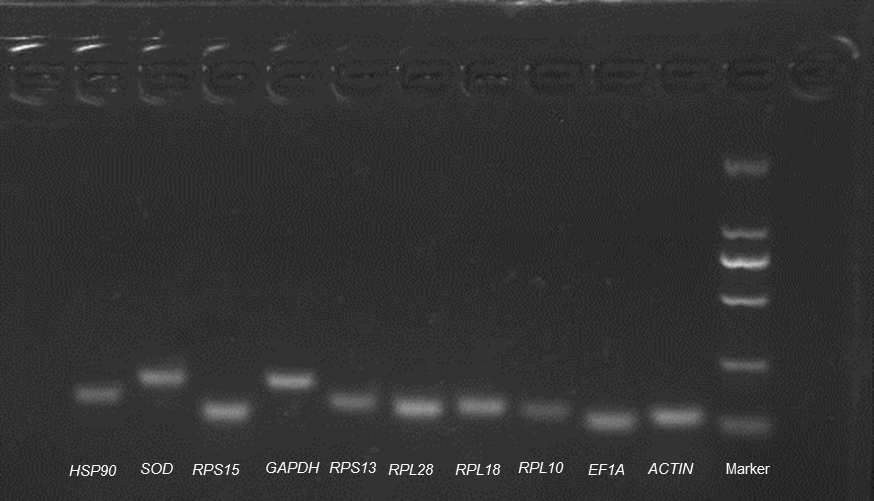

Supplement: S1 Fig — (TIF) [file pone.0192521.s001.tif]

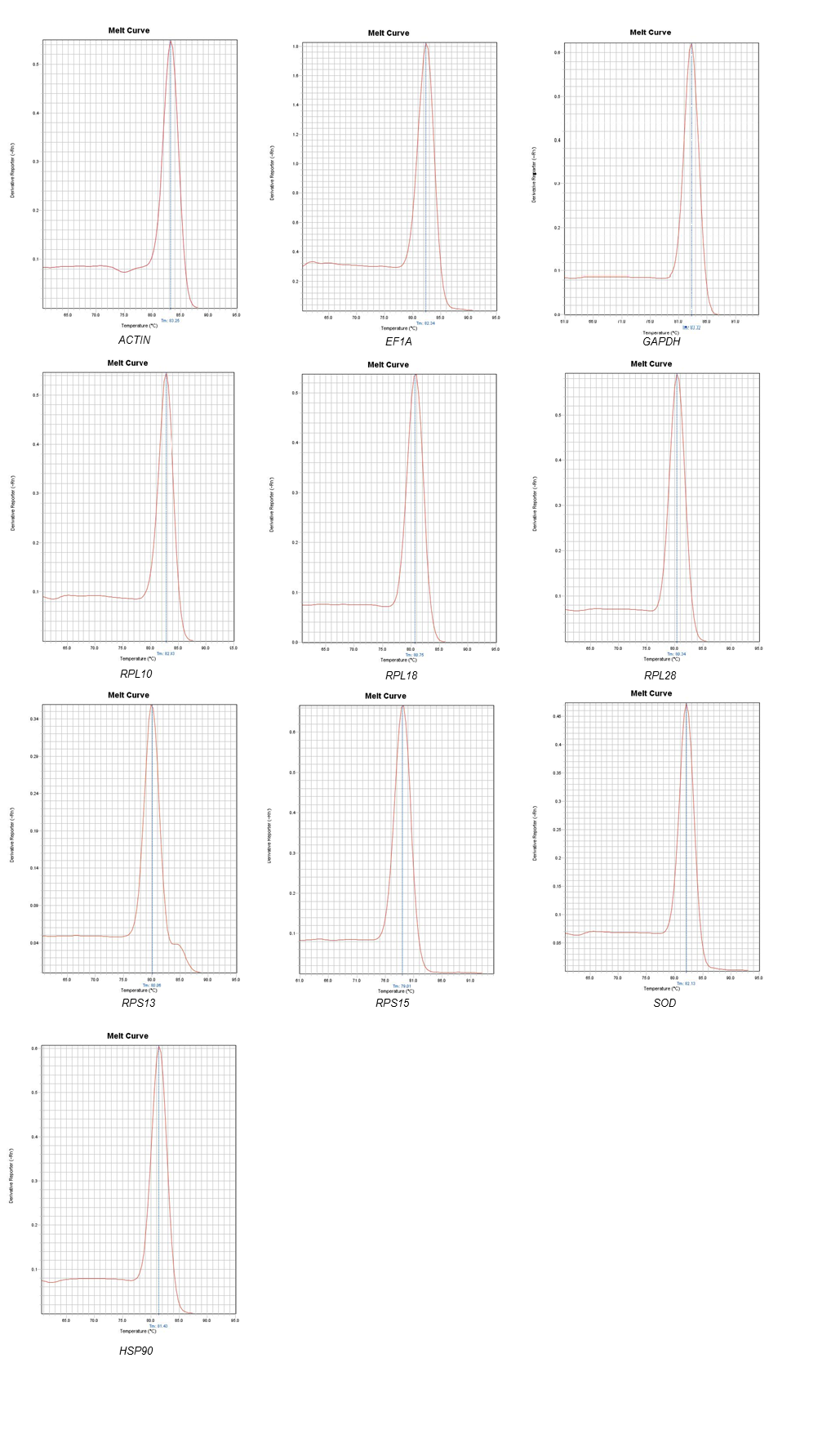

Supplement: S2 Fig — (TIF) [file pone.0192521.s002.tif]
